# Insecticide-treated eave nets and window screens for malaria control in Chalinze district, Tanzania: a study protocol for a household randomised control trial

**DOI:** 10.1186/s13063-022-06408-4

**Published:** 2022-07-19

**Authors:** Olukayode G. Odufuwa, Sarah Jane Moore, Zawadi Mageni Mboma, Emmanuel Mbuba, Joseph Barnabas Muganga, Jason Moore, Rose Philipo, Mohammed Ally Rashid, Rune Bosselmann, Ole Skovmand, John Bradley

**Affiliations:** 1 Vector Control Product Testing Unit, Ifakara Health Institute (IHI), Bagamoyo, Tanzania; 2 Vector Biology Unit, Swiss Tropical and Public Health Institute (SwissTPH), Allschwil, Switzerland; 3 University of Basel, Basel, Switzerland; 4 MRC International Statistics and Epidemiology Group, London School of Hygiene and Tropical Medicine (LSHTM), London, UK; 5 Vegro Aps, Copenhagen, Denmark; 6 MCC47, Montpellier, France

**Keywords:** Randomised controlled trial, House modification, Malaria, Insecticide-treated nets, Mosquito, Eaves, Windows, Vector-borne diseases, Tanzania

## Abstract

**Background:**

Long-lasting insecticidal nets (LLINs) have contributed to the reduction of malaria in sub-Saharan Africa, including Tanzania. However, they rely on daily user behaviour and high coverage which is difficult to maintain. Also, insecticide resistance among malaria vector mosquitoes is contributing to reduced efficacy of control tools. To overcome these problems, we propose to evaluate a new tool for house modification, the insecticide-treated eave nets (ITENs) in combination with insecticide-treated window screens (ITWS) incorporated ﻿with dual active ingredient (dual AI) for the control of malaria.

**Methods:**

Four hundred and fifty (450) households with intact walls, open eaves without screens or nets on the windows in Chalinze district will be eligible and recruited upon written informed consent. The households will be randomly allocated into two arms: one with ITENs and ITWS installed and the other without. Malaria parasite detection using a quantitative polymerase chain reaction (qPCR) will be conducted shortly after the long rain (June/July, 2022) as the primary outcome and shortly after the short rain (January/February, 2022) as the secondary outcome. Other secondary outcomes include clinical malaria cases, and density of malaria vectors and nuisance after the short rain and long rain. In addition, surveys will be conducted in households with ITENs and ITWS to estimate the intervention’s cost during installation, adverse effects one month after installation, and presence, fabric integrity and user acceptance six and twelve months after installation. Bioefficacy and chemical content will be evaluated twelve months after installation.

**Discussion:**

ITENs and ITWS have been shown in Kenya to reduce indoor mosquito density. However, it is not known if indoor mosquito density reduction translates into reduction of malaria cases. Data from the study will measure the potential public health value of an additional intervention for malaria control at the household level in areas of mosquito insecticide resistance that does not require daily adherence.

**Trial registration:**

The study is registered on ClinicalTrials.gov.

**Supplementary Information:**

The online version contains supplementary material available at 10.1186/s13063-022-06408-4.

## Administrative information

The order of the items has been modified to group similar items (see http://www.equator-network.org/reporting-guidelines/spirit-2013-statement-defining-standard-protocol-items-for-clinical-trials/). The numbers in {brackets} in this protocol refer to SPIRIT checklist item numbers.

**Table:** 

Title {1}	Insecticide treated eave nets and window screens for malaria control in Chalinze district, Tanzania: a study protocol for a household randomised control trial.
Trial registration {2a and 2b}	Registry name: ClinicalTrials.gov Trial Identifier: NCT05125133 https://clinicaltrials.gov/ct2/show/NCT05125133
Protocol version {3}	4^th^ November, 2021. V06
Funding {4}	Medical Research Council Joint Global Health Trials MR/T003677/1.
Author details {5a}	Olukayode G. Odufuwa: Ifakara Health Institute (IHI), Tanzania; Swiss Tropical and Public Health Institute (Swiss TPH); University of Basel; London School of Hygiene and Tropical Medicine (LSHTM), London. Sarah Jane Moore: Ifakara Health Institute (IHI), Tanzania; Swiss Tropical and Public Health Institute (SwissTPH), Switzerland; University of Basel, Basel, Switzerland. Zawadi Mageni Mboma: Ifakara Health Institute (IHI), Tanzania; London School of Hygiene and Tropical Medicine (LSHTM), London. Emmanuel Mbuba: Ifakara Health Institute (IHI), Tanzania; Swiss Tropical and Public Health Institute (SwissTPH), Switzerland; University of Basel, Basel, Switzerland. Joseph Barnabas Muganga: Ifakara Health Institute (IHI), Tanzania. Jason Moore: Ifakara Health Institute (IHI), Tanzania; Swiss Tropical and Public Health Institute (SwissTPH), Switzerland; University of Basel, Basel, Switzerland. Rose Philipo: Ifakara Health Institute (IHI), Tanzania. Rune Bosselmann: Vegro Aps, Copenhagen, Denmark; Ole Skovmand: Intelligent Insect Control, Montpellier, France; John Bradley: London School of Hygiene and Tropical Medicine (LSHTM), London.
Name and contact information for the trial sponsor {5b}	Ifakara Health Institute (IHI), Plot 463, Kiko Avenue Mikocheni. P.O. Box 78 373, Dar es Salaam, Tanzania. Telephone: +255 222 774756 Email: info@ihi.or.tz
Role of sponsor {5c}	Sponsor approved the study design; data collection, data management, analysis plan and protection of human participants through Ifakara Health Institute Institutional review board (IHI-IRB). No ultimate authority exists between the sponsor and funding parties. The funding party was only involved in supplying funds for the implementation of the trial.

## Introduction

### Background and rationale {6a}

Long-lasting insecticidal nets (LLINs) have contributed to the large reduction of malaria globally, particularly in sub-Saharan Africa in the last decade [1]. However, malaria reduction is stalled or reversed in many malaria-endemic countries, including Tanzania. Reasons for this include increasing insecticide resistance of malaria vectors [2], low access to LLIN [3], LLINs not lasting up to three years so they wear out before the next mass distribution [4] and insufficient compliance with daily LLIN use [5]. Therefore, additional cost-effective and long-lasting vector control tools are required that will confer protection against insecticide-resistant malaria vectors and that protect every member of the household with minimal user compliance.

**Fig. 1:**
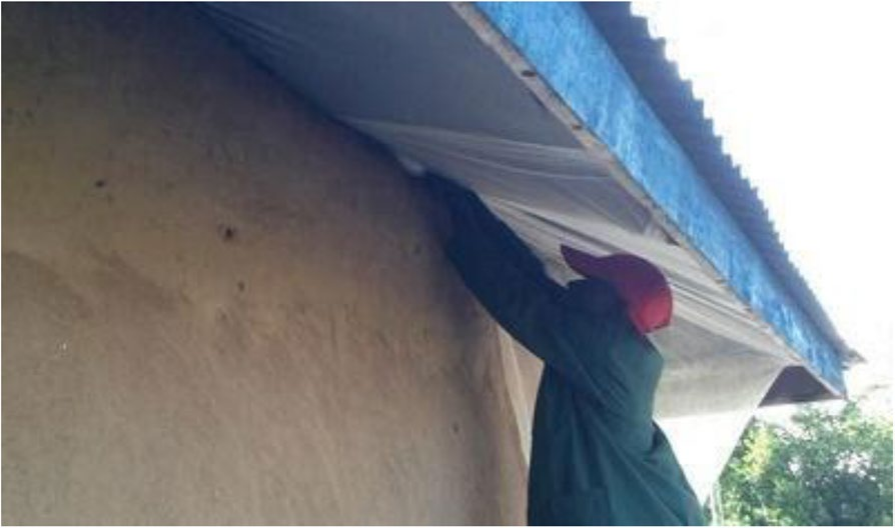
ITENs being installed. The net is provided on a roll and takes only thirty min to install, but lasts more than 4 years in Kenya

The vector control tool for evaluation in this trial is the insecticide-treated eave nets (ITENs) in combination with insecticide-treated window screens (ITWS) incorporated with a dual active ingredient (dual AI) for house modification (Fig. 1). These can protect everyone in the house by blocking mosquito entry and provide community protection by killing mosquitoes that encounter them. The tool is incorporated with deltamethrin insecticide and piperonyl butoxide (PBO) synergist, both are found in insecticide-treated nets that are already in use among the population to control pyrethroid-resistant mosquitoes [6]. A great advantage is they can be used in combination with other AIs and can be used with AIs that cannot be used on bed nets, because they do not come into daily contact with users. The netting is supplied on a roll and, as shown in a study in western Kenya, can be easily applied to houses using locally available tools in just thirty min per house [7]. The pilot study in Kenya used pyrethroid only ITENs and ITWS among eighty households and showed a 75% reduction of vector densities in the houses using ITENs, ITWS and LLINs when compared to households using LLINs only [7].

This protocol describes the activities that will be implemented in a trial designed to investigate the efficacy of ITENs with ITWS on malaria parasite prevalence measured by quantitative polymerase chain reaction (qPCR), and other secondary outcomes.

### Objectives {7}

The primary objective of the trial is to investigate the efficacy of ITENs with ITWS on malaria parasite prevalence among residents aged six months and above, measured by quantitative polymerase chain reaction (qPCR) shortly after long rainy season (June/July 2022), and after the short rainy season (January/February 2022) as a secondary outcome. Other secondary objectives are to estimate the effect of the interventions on clinical malaria cases, and density of malaria vectors (*Anopheles gambiae* sensu lato and *An. funestus* sensu lato) and nuisance (*Culex quinquefasciatus*) mosquitoes after the (i) short rainy season (January/February 2022) and (ii) long rainy season (June/July 2022). The cost (fabric amount and time duration) of installing the intervention, perceived adverse effects one month after installation, and physical durability and community acceptance of the tool six months and twelve months after installation, as well as chemical durability twelve months after installation.

### Trial design {8}

**Fig. 2:**
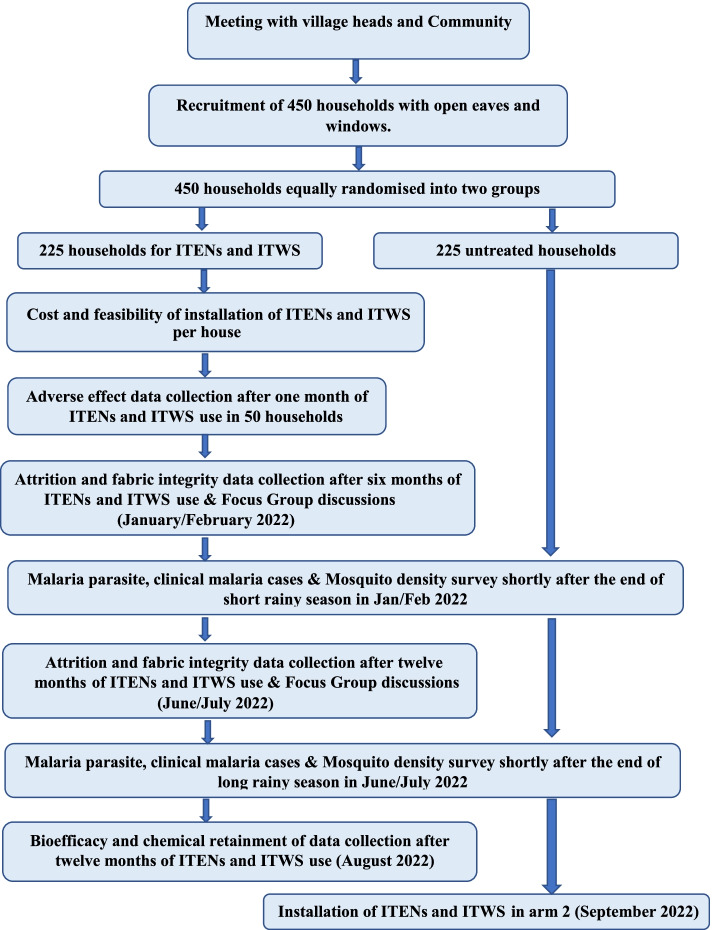
Trial framework

This is a household randomised superiority trial of households with ITENs and ITWS installed, compared to those without them, among villages in Chalinze district located in Pwani region, Tanzania (Fig. 2). Four hundred and fifty households will be randomly allocated equally into two arms and will be surveyed twice in the subsequent year: short rainy season (January/February 2022) and long rainy season (June/July 2022). The surveys will assess the malaria prevalence of all participants using quantitative polymerase chain reaction (qPCR) [8]; clinical malaria cases defined by fever and malaria rapid diagnostic test (mRDT), SD Bioline; and the density of malaria vectors and nuisance mosquitoes will be measured using Centres for Disease Control (CDC) light traps [9].

In addition, a structured questionnaire will be administered to obtain the estimated (i) time and material cost of installing the tool (feasibility) during installation, (ii) perceived adverse effects after one month after installation and (iii) community acceptance of ITENs with ITWS. The physical durability of the intervention will be evaluated through physical inspection of the ITENs and ITWS for the continued presence and fabric integrity. Chemical durability will be assessed using samples of netting that will be evaluated for bioefficacy using laboratory-reared populations of pyrethroid-resistant *An. arabiensis,* *Cx. quinquefasciatus*, *An. funestus* and pyrethroid susceptible *Aedes aegypti* mosquitoes, followed by chemical testing according to standard WHO guidelines and procedures [10]. In addition, the perception, use and practice of the intervention will be examined through focus group discussions (FGDs).

## Methods: participants, interventions and outcomes

### Study setting {9}

The trial will be implemented in the villages of Pongwe, Madesa and Mazizi in Chalinze district located in coastal Tanzania. The average rainfall is 1200 to 2100 mm per year and the average temperature is approximately 28 °C in the region. The region has two rainfall seasons yearly: the long rainfall from March to May and short rainfall from November to December. The main occupation for majority of the residents in villages of Chalinze is farming. The population of the study area was 13,740 while the average household size in the district was 4.5 [11]. The malaria prevalence by PCR in the study area was more than 40% in the survey designed to assess gametocyte in children of age 7 years old and above (Lorenz Hofer. *pers comm.*). More than 60% of houses had window screens and 13% had closed eaves in a study conducted previously in the study area [11]. An estimated 1800 people based on the average household size and number of households will be tested for malaria at each survey; thus, nine hundred people per arm will be screened for malaria.

### Eligibility criteria {10}

Recruitment for the trial will be by household. To be eligible, residents must live in a house with strong (intact) walls, having open eaves and unscreened windows. Residents of study houses who are over the age of six months will be eligible for the follow-up surveys.

#### Eligibility criteria for individuals who will perform the interventions

Carpenters required to install the ITENs and ITWS, and trained community technicians will be recruited from the participating villages as they are most familiar with the house structures and location. Technicians will (i) collect information on the feasibility cost of installing ITENs and ITWS, (ii) collect mosquitoes with CDC light traps, (iii) assess attrition, fabric integrity, bioefficacy, chemical retainment and (iv) assess community practice and acceptance of ITENs and ITWS installed in houses. Nurses/clinical officers or clinicians that will withdraw blood samples from household members for qPCR analysis and RDTs will also be recruited from the study villages. The purpose of recruiting these individuals from the villages is to utilise their local knowledge and community trust of these individuals as well as to contribute to building the capacity and economy of the villages where the trial will be conducted.

### Who will take informed consent? {26a}

Written informed consent in the local language, Kiswahili, will be voluntarily obtained by trained field interviewers recruited from the villages. Informed consent will also be read aloud to non-literates in Kiswahili in the presence of a witness, after which the participant will be asked to mark a thumb impression on the form and the witness will be asked to sign to obtain acceptance of participation. Consent for household participation will be sought from heads of households that are eighteen years of age and above. Additional written informed consent will be sought from individuals prior to malaria testing. Participants under eighteen years old will be recruited for malaria testing upon combination of written informed consent from parent/guardian and assent from the child based on the age. A different assent form will be provided to children aged between seven to twelve years old and those between thirteen to seventeen years old (Additional file 1).

### Additional consent provisions for collection and use of participant data and biological specimens {26b}

Description of use of blood for malaria diagnostic using RDT and qPCR is explained in the written informed consent. Samples will be used only for malaria screening, and no samples will be stored for further tests.

## Interventions

### Explanation for the choice of comparators {6b}

The control arm will consist of houses satisfying the eligibility criteria and that do not receive the intervention. To estimate the additional protection of the intervention given the current malaria control tool in use in the population, houses in the same location without the intervention is ideal.

### Intervention description {11a}

#### Intervention group

The intervention is hardwearing insecticide-treated netting that is used to cover the eaves (gaps between the wall and roof) of houses and to screen windows. The insecticides incorporated in the ITENs and ITWS yarn of 0.152 mm in diameter are deltamethrin at 3 g AI/kg, which corresponds to 144 mg/m^2^ and PBO synergist at 10 g/kg which corresponds to 480 mg/m^2^, as used in the so-called dual-AI LLIN or “resistance breaking” nets [6]. The nets were manufactured by Moon Netting FZCO, United Arab Emirates. The combinations of deltamethrin and PBO synergist have been shown to be efficacious against pyrethroid-resistant Anopheles mosquitoes and malaria [12]. These nets are supplied on a roll and attached to the wood around the roof, frame of the windows and on wood between walls using staple guns and hammer. The nets will be used to cover all obvious mosquito entry points including opened eaves, windows and holes in house walls that are greater than or equal to 10 cm diameter.

### Criteria for discontinuing or modifying allocated interventions {11b}

Participants are free to withdraw from the study, and they will be given the option to either keep the intervention or uninstall the nets from the eaves and windows. If perceived unacceptable adverse effects related to the intervention are reported by the participants, the intervention shall be removed upon the request of the participants by the study team, as referred in the Informed Consent form (Additional file 1).

### Strategies to improve adherence to interventions {11c}

ITENs and ITWS are hardwearing insecticide-treated netting, thus minimal risk of non-adherence. Houses allocated to the negative arm will have ITENs and ITWS installed at the end of the trial.

### Relevant concomitant care permitted or prohibited during the trial {11d}

At each survey, clinical officers/nurses/clinicians will withdraw blood samples from all participants with an axillary temperature of 37.5° or over for malaria parasites using a SD Bioline Malaria Ag Pf/Pan rapid diagnostic test (RDT) for point of care as per Tanzania Ministry of Health guidelines [13], and to establish clinical malaria cases. Any participant (s) that tests positive using mRDT will be treated using Artemether Lumefantrine (ALu). This drug is approved by the national guideline for treatment of uncomplicated malaria [13].

### Provisions for post-trial care {30}

ITENs and ITWS will be installed in houses randomly selected in the negative control arm at the end of the trial to ensure that all groups have access to the intervention.

### Outcomes {12}

#### Primary outcome

Prevalence of malaria measured by qPCR shortly after long rainy season (June/July, year 2022) in households with ITENs and ITWS installed in comparison to those without.

#### Secondary outcomes

- Prevalence of malaria measured by qPCR shortly after the short rainy season (January/ February 2022) in households with ITENs and ITWS installed in comparison to those without.
- Prevalence of clinical malaria cases (defined by axillary temperature of 37.5° or above and positive mRDT) shortly after the short rainy season (January/February 2022) and long rainy season (June/July 2022) in households with ITENs and ITWS installed in comparison to those without.
- Density of malaria vectors and nuisance mosquitoes present in houses with ITENs and ITWS installed in comparison to the houses without after the short (January/February 2022) and long (June/July 2022) rainy season measured by CDC light trap.
- The amount of fabric and time cost of installing ITENs and ITWS per house.
- The percentage of adverse effects among technicians and houses with ITENs and ITWS one month after installation.
- The physical presence and numbers of holes visually observed in ITENs and ITWs six months and twelve months after installation.
- The bioefficacy of ITENs and ITWs twelve months after installation measured by percentage knockdown after one hour and mortality of laboratory-reared mosquitoes at twenty four hours after exposure.
- The chemical content of ITENs and ITWs after twelve months after installation.
- The percentage acceptance of ITENs and ITWS in the community using the structured questionnaire.
- The behaviour, practice and use of ITENs and ITWS in the community from FGDs.

#### Participant timeline {13}

Participant timeline is shown below.

**Table:** 

		STUDY PERIOD						
	Enrolment	Allocation	Post allocation					Closeout
TIMEPOINT	May	June, 2021	Aug, 2021	Dec, 2021	Jan, 2022	June, 2022	July, 2022	Sept, 22
Eligibility screen/ baseline data	X							
Informed consent /	X							
Allocation								
Installation in the intervention arm		X						
Installation in the control arm								X
ASSESSMENTS								
Perceived adverse effect			X					
500 μl blood from household members of above 6 months old					X	X		
Density of malaria vectors and nuisance mosquitoes					X	X		
Feasibility Cost		X						
Longevity, fabric integrity, community acceptance and practice.				X		X		
Bioefficacy and chemical durability						X		

### Sample size {14}

With 225 houses per arm was an assumed 4.5 residents per household [14], then if prevalence in the control arm is 20% the study would have 90% power to detect a relative reduction of 30% to a prevalence of 14% assuming a conservative between-house coefficient of variation of 0.5.

### Recruitment {15}

Once permission from the District Medical Officer and village heads is obtained, meetings with the village leaders will be convened to discuss the aim and procedures of the trial, and to select the villages where houses that meet eligibility criteria can be found. Once villages are selected, community sensitisation will be conducted among all the selected villages to provide information on the purpose of the study to the people, including the adverse consequences of vector-borne diseases, the benefits and risks of using ITENs and ITWS, and caring for the intervention. This will be followed by recruitment of the households through door-to-door process, facilitated by community leaders as key informants to introduce field teams to households.

## Assignment of interventions: allocation

### Sequence generation {16a}

Household identification numbers stratified by sub-village will be provided to a statistician that will not be in the field during the community enrolment. Simple randomization within blocks of sub-villages to randomly allocate households between two arms will be conducted. Randomisation of all study households will be done simultaneously.

### Concealment mechanism {16b}

This trial will not have continuous recruitment. All households will be recruited at the start of the study and randomised simultaneously.

### Implementation {16c}

After enrollment of all households by trained field workers, the trial statisticians will generate allocation sequence and assign households to interventions through randomisation stratified by sub-village.

## Assignment of interventions: blinding

### Who will be blinded {17a}

This is an open label trial because the intervention can be visually observed.

### Procedure for unblinding if needed {17b}

The design is open label so unblinding will not occur.

## Data collection and management

### Plans for assessment and collection of outcomes {18a}

Quantitative data such as malaria prevalence, clinical malaria cases, mosquito density, feasibility, cost, adverse effects, attrition, integrity and use practice of the intervention will be entered using Open Data Kit (ODK) [15] installed on android tablet computers. Paper data forms will be available to the field team for use in the eventuality of tablet computer failure. Data collected will be uploaded to the IHI secure data repository for data analysis. Qualitative data will be audio recorded and transcribed [16]. Interviews will be saved in an external hard-drive and locked in a secure file cabinet. Mosquitoes will be collected using CDC light traps to measure the effect of the intervention on the indoor density of malaria vectors and nuisance mosquitoes [9]. Blood samples will be collected using New MiniCollect® K2EDTA Capillary Tube, manufactured by Greiner Bio-One, Austria. The blood samples will be kept in cool box before they are transferred to the lab for qPCR analysis [8]. To assess clinical malaria cases, calibrated ear thermometer will be used to record body temperature of those with positive mRDT. The study personnel, field workers/data collectors are trained on correct data input methods to ensure data integrity.

### Plans to promote participant retention and complete follow-up {18b}

Field interviewers recruited to support data collection will be from the study villages. Phases of data collection will involve door-to-door surveys, with respective sub-village heads guiding field interviewers to households, as participants are more likely to stay in the trial when familiar faces are involved. Achieving complete follow-up in village settings may also be influenced by time of data collection, as most of the study village residents are farmers and are more likely to be in their farms throughout the daytime during cultivation and harvesting season; therefore, follow-up visits for data collection will occur at a time when participants are available, e.g. in the early morning or evening. Data collectors will revisit absent households as much as possible to ensure completeness of data collection. However, in case of discontinuation by participants, the information that will be recorded are date, data collectors name, house code, GPS location and consent.

### Data management {19}

Data collected using the ODK application installed on android tablets will be uploaded onto the IHI secured server and downloaded for analysis. Data collected using paper forms will be double entered into a computer using double-key entry methods to facilitate cross-referencing and validation. The two sets of entries will be compared, and any discrepancies found between the two databases will be resolved by checking the data forms as per analysis plan. Audio recordings and transcriptions, as well as notes taken during the focus group discussions will be stored securely. The data, both in hard copy and digital format, will be kept for the purpose of analyses until consideration and clearance of the final report.

Research records for all study participants including history, physical findings, and laboratory data are to be maintained in a secure storage facility for 10 years or until notified by grantee as per Tanzanian ethical guidelines. The grantee will be notified in writing and acknowledgment must be received prior to destruction or relocation of research records. All raw and cleaned data forms will be archived in the project office in a dedicated filing cabinet and all data files will be retained on the IHI central data server in accordance with IHI and Tanzania National guidelines.

### Confidentiality {27}

All study-related information will be stored securely at IHI. Data collectors are trained on maintaining confidentiality of study participants’ information. Households and participants will be given unique identification numbers; thus, participant’s study personal information will be anonymized before analysis or if data will be shared based on data sharing agreement. Personal information will only be shared based on written permission of the participant, for purposes of independent monitoring among representatives of either government and regulatory authorities, or site Institutional Review Board committees/Ethics Committees.

### Plans for collection, laboratory evaluation and storage of biological specimens for genetic or molecular analysis in this trial/future use {33}

Trained clinical officers/nurses/clinicians will screen for malaria parasites among all residents of study households by withdrawal of no more than 500 μl of blood in Eppendorf tubes that will be transferred to the laboratory at IHI, Kingani for detection of DNA of Plasmodium species [8] using quantitative polymerase chain reaction (qPCR) analysis. Point of care diagnosis for febrile individuals will be SD Bioline Malaria Ag Pf/Pan rapid diagnostic test (RDT) as per Tanzanian guidelines [13].

Used RDT kits will be stored in a locked shipping container at IHI until the end of the study, then the samples will be destroyed on-site in a high-temperature incinerator.

## Statistical methods

### Statistical methods for primary and secondary outcomes {20a}

The primary endpoint which is detection of malaria parasitemia through qPCR after the long rainy season (June/July 2022) will be analysed using mixed effects binary logistic regression, with a random effect for household to account for clustering. Analysis of secondary endpoints qPCR confirmed malaria parasitemia after the short rainy season (January/February 2022) as well as clinical malaria cases and malaria prevalence shortly after the short rainy season and long rainy will also be analysed following the same approach.

Negative binomial mixed effect model will be performed for mosquito density data, with random effect for household.

The baseline characteristics collected during the survey, including age, sex, education, number of participants, household size, occupation, sleeping spaces, LLINs per house, nature of houses (wall, roof and floor), community acceptance of LLINs (use rate and any side effects) and socioeconomic quintiles will be presented by arm.

Principal component analysis (PCA) will be carried out to determine a combination of variables for socioeconomic status to explain the overall observed variation and reduce the complexity of the data, by calculating a weighted score for the socioeconomic status of each household of the population and divided into five quintiles: lowest, low, middle, high and highest.

The results of the proportional outcomes (attrition, fabric integrity and community acceptance) will be presented in percentages.

Recorded information during the FGD will be transcribed and grouped into different themes based on inductive thematic analysis of interview summaries. Reports will be illustrated with verbatim quotes of themes and sub-themes identified.

### Interim analyses {21b}

No interim analyses will be carried out.

### Methods for additional analyses (e.g. subgroup analyses) {20b}

An analysis of the intervention effect within subgroups defined by age (under 5s, aged 5–15 and aged 16 or over), socioeconomic status and gender, among others, will be carried out on malaria prevalence at both surveys using the same methods as for the primary endpoint.

### Methods in analysis to handle protocol non-adherence and any statistical methods to handle missing data {20c}

Minimal protocol non-adherence is expected because of the nature of the intervention; however, we will carry out an intention to treat in which households are analysed according to allocation regardless of whether the intervention was adhered to. A secondary per protocol analysis which excludes houses that removed the intervention will be carried out. At an individual level, participants who report being away from home for more than a week during the fortnight preceding testing will be excluded from the primary analysis but included in a secondary analysis. We expect minimal missing data on the primary endpoint, in which case complete case analysis will be used. If there is more missing data than expected, a secondary analysis in which multiple imputation is applied under the assumption of missing at random will be carried out.

### Plans to give access to the full protocol, participant-level data and statistical code {31c}

Full protocol, statistical analysis plan, anonymised level data and statistical code will be made available on reasonable request.

## Oversight and monitoring

### Composition of the coordinating centre and trial steering committee {5d}

The Ifakara Health Institute is the trial sponsor and coordinating centre. Study investigators meet weekly to review trial progress. A trial steering committee consisting of study investigators and independent experts will meet after every study survey.

### Composition of the data monitoring committee, its role and reporting structure {21a}

Although we do not anticipate any safety issues in this trial since the intervention is similar to LLINs which are used extensively in malaria-endemic regions, a data monitoring committee has been appointed. The committee is composed of a clinician, an entomologist and a statistician, is independent of the trial sponsor and funder, and has no competing interests. The committee will meet after the data from each survey have been collected. The committee will consider safety data and data on trial endpoints and make recommendations on whether the trial should continue.

### Adverse event reporting and harms {22}

The assessment of the risk to humans by the World Health Organization (WHO) [17] is that no unacceptable exposures were found in maintenance and use of LLINs incorporated or coated with deltamethrin and PBO synergist and that washing or sleeping under them does not pose undue risk to adults. Moreover, there is reduced contact with ITENs and ITWS. Nevertheless, participants will be advised on possibilities of adverse effects. In case of serious adverse effect related to the use of the study items, although not expected, affected participants will be provided with free medical care.

An assessment of adverse effects will be made using a questionnaire given in Additional file 1 and will be administered to carpenters and technicians as well as fifty randomly selected households in the village at four weeks post-installation of ITENs and ITWS. The households will be selected by the statistician using a simple random selection procedure blocked at a sub-village level. The Principal Investigator shall inform the Bagamoyo District Medical Officer and the IHI ethical review board about possible reporting of adverse effects of use of ITENs and ITWS by the participants.

### Frequency and plans for auditing trial conduct {23}

No formal trial audit is anticipated. Study investigators meet weekly to review trial conduct. The independent (Data Safety Monitoring Board) DSMB meets after each of the study surveys.

### Plans for communicating important protocol amendments to relevant parties (e.g. trial participants, ethical committees) {25}

Any protocol amendments and/or deviations will be fully justified and documented and agreed upon by the sponsor, study investigators, and village representatives. Application for such protocol amendment will be written to the ethics committee and for clearance. If approval is attained from the ethics committee, the changes to the protocol will be communicated to the participants via village leaders and during study follow-up.

### Dissemination plans {31a}

Data will be disseminated by report to the sponsor, followed by peer-reviewed publication targeted towards scientists and policy-makers. We will publish the results and data sets in open-access, indexed, peer-reviewed journals, making the findings and the anonymised data available to all stakeholders at reasonable requests. Data obtained from the study will be presented at international conferences and stakeholder meetings including the National Malaria Control Program. At the end of the project a meeting will be held to update the local community and the District Medical Officer (DMO), to present findings and answer any questions arising.

## Discussion

For malaria control, demonstrating the effectiveness of ITENs and ITWS would bring many benefits. ITENs and ITWS have the potential to be long-lasting, cost-effective and should protect everybody in the household at all times when they are indoors. The interventions can be fitted to any house style, unlike traditional house screening that is costly and better suited to improved housing designs. Unlike LLINs, they require no compliance from the user. Data from a pilot study in Kenya indicate that ITENs/ ITWS are far more cost-effective than LLINs, costing USD 1–1.75 less per household per year (assuming 3 nets and 6 people per household) due to lower material costs and improved longevity, and the saving is even greater compared to indoor residual spraying. In a separate study, the efficacy of ITENs and ITWS in the Semi Field System (SFS) setting against dengue vectors will be examined, and it is likely that ITENs and ITWS could work against other vector-borne diseases such as filariasis. It is desirable as a means of preventing nuisance mosquito bites, which will likely improve uptake of the intervention. ITENs and ITWS are good candidates for deployment of new insecticides as they are not washed or in close contact with users and the fast knockdown characteristic of pyrethroids may not be required. This may offer great advantages in insecticide resistance management, as different insecticides to those applied to LLINs can be used in further iterations of the tool following proof of efficacy. There may also be an economic boost to low-income countries that use ITENs and ITWS. Approximately 85% of the jobs associated with LLIN production are in cutting and sewing activities (fabric to finished LLIN). Since the material for ITENs and ITWS is sent on rolls and installation occurs at the site of implementation, there is a transfer of employment from the manufacturing site to the malaria-endemic country.

A limitation of this trial is that randomisation is at the household level rather than the village level. This means the trial will only capture direct effects of the intervention and not capture indirect effects so-called “mass-effect” [18]. A trial in which villages are randomised would be substantially larger than the present trial and require much greater funding and resources. Nevertheless, this trial will gather valuable data on the efficacy of this promising intervention against both epidemiological and entomological endpoints.

### Trial status

The trial protocol version number is 6 dated 4 November 2021. Randomisation of households to study arm completed in July 2021 and in August 2021 installation of the intervention was completed for households in the intervention arm. Recruitment of individuals for the first malaria prevalence survey took place in January 2022 and for the second will take place in July 2022.

## Supplementary Information

**Additional file 1: Appendix 1**. Informed consent forms.

## Data Availability

The sponsor and funder will have access to the final trial dataset. The final trial anonymised data for this protocol can be supplied on reasonable request.

## Abbreviations

Alu: Artemether lumefantrine
AI: Active ingredient
ATP: According to protocol
CI: Confidence interval
CRF: Case Report Form
DMO: District Medical Officer
DSMB: Data Safety Monitoring Board
GLP: Good Laboratory Practice
HIN: Household Identification Number
IHI: Ifakara Health Institute
IRB: Institutional Review Board
ITENs: Insecticide-Treated Eave Nets
ITWS: Insecticide-Treated Window Screens
LLIN: Long-Lasting Insecticidal Net
LTFU: Loss To Follow-Up
PCR: Polymerase Chain Reaction
PI: Principal Investigator
RCT: Randomised Controlled Trial
RDT: Rapid Diagnostic Test
SAE / AE: Serious Adverse Events / Adverse Events
SOP: Standard Operating Procedure
UIC: Unique Identifier Code
