# Supplementary material for: Insecticide-treated eave nets and window screens for malaria control in Chalinze district, Tanzania: a study protocol for a household randomised control trial
Source: Trials. 2022 Jul 19;23:578. doi: 10.1186/s13063-022-06408-4 (PMC9295261; doi:10.1186/s13063-022-06408-4)
Supplement: Supplementary file 1 — Additional file 1: Appendix 1. Informed consent forms. [file 13063_2022_6408_MOESM1_ESM.docx]

**Appendix 1: Informed Consent Forms**

**INFORMED CONSENT FORM FOR HOUSEHOLDERS**

**Name of Principle Investigator:** Dr. Zawadi Mageni Mboma

**Name of Organization:** Ifakara Health Institute, Bagamoyo, Tanzania

**Name of Sponsor:** London School of Hygiene and Tropical Medicine (LSHTM)

Household ID No. |__|__|__|__|

**Part 1: Information sheet for households**

**Feasibility and community acceptability of Insecticide Treated Eave Nets and Insecticide Treated Window screens in Tanzania.**

**Introduction**

My name is …………………...<name of ICF administrator>. I am working for the Ifakara Health Institute, Tanzania. We are here to investigate the efficacy, community acceptability and the ease of installing a new product that is designed to prevent mosquitoes from entering the house. The name of the product is Insecticide Treated Eave Nets (ITENs) with Insecticide Treated Window screens (ITWS), they are basically nets treated with special insecticide in the factory that do not need retreatment throughout their functional life, the insecticide in them, kills mosquitoes. These nets are designed for installation on windows and eaves, to block all mosquito from entry. Therefore, preventing you and your household members from malaria and other vector-borne diseases.

This study is meant to demonstrate if ITENs and ITWS are efficacious in controlling local strains of mosquitoes and to know the cost of installing them and if people will be willing to use them if provided for public use.

Your village has been selected for this study. We have informed the district and village leaders about the study and permission has been granted. ITENs with ITWS will be installed in your house to cover your eaves and windows to protect you and your house members from mosquito bites and to kill mosquitoes, and at the same time allowing airflow.

To understand how good these tools are in your community, we shall install ITENs with ITWS in your house, we will measure the time and economic cost it takes to install them, and we will also ask you about the tools, whether you think they are beneficial for malaria and other vector-borne diseases control and how they can be improved on for public use. We will also conduct a malaria test for you and members of your household.

**Installation of ITENs and ITWS will begin immediately you agree to participate in the study, you shall provide information about your household, and after 6 months and one year, we will visit you again. We shall request for permission to come in your house to look at the condition of the ITENs with ITWS, we shall ask questions about the condition. Shortly before the end of each of the two rainy seasons from now (short rainy season in year 2021 and long rainy season in year 2022), we shall come to place CDC light traps in your house to capture mosquitoes. Also, after each of the two rainy seasons (short rainy season in the year 2021 and long rainy season in the year 2022), we shall come to conduct malaria test for you and the members of your household. Two tests will be done, that include, RDT and dry blood spots. All these tests will involve pricking of a finger. The pricking for all these tests will only be done once. The blood will be stored for 10 years at IHI, Bagamoyo for malaria testing only, and will not be used for another test.**

**If you agree to participate, below are the research procedures:**

1. Head or any adult member of your household will be interviewed and questions about your household’s demographic and socioeconomic characteristics, house structures, sleeping arrangement, pattern of use of nets and perception on ITENs with ITWS will be asked.
2. We shall install ITENs with ITWS in your house to cover the windows and eaves.
3. The team shall visit again after one month of ITENs with ITWS installation, to interview you and your household members on experiences with the presence of ITENs and ITWS in your house.
4. After 6 months and one year of installation, the team shall visit again to check for the presence and condition of the ITENs with ITWS and you shall also be interviewed again. You or any other adult family member shall be quizzed on acceptance of the tool, handling, use and perception on the tool.
5. The interview may last about 30 minutes.
6. **Shortly before the end of the rainy seasons, we shall place CDC light traps in your household to collect mosquitoes.**
7. We shall also test all the house members for malaria shortly **after the end of 1) long rainy season, 2021, 2) short rainy season 2021 and 3) long rainy season 2022.**
8. In addition, you may also be selected to participate in a focus Group Discussion about your experience on the use of ITENs and ITWS, this will also last about 30 minutes.

**Risks and adverse effects**

Sleeping in houses with ITENs and ITWS will protect you from mosquito bites indoor and diseases such as malaria and dengue. However, it does not protect when you and your family are not inside the house, so it is possible you still get bitten by mosquitoes and eventually be infected with malaria. Thus, if you suffer from fever, you should immediately approach the health staff at the Government dispensary for treatment where adequate facilities exist for treatment of malaria. You may seek advice/assistance from the Ifakara Health Institute as per the contact details given below.

Deltamethrin and PBO, the insecticide used on the nets and screens, have been tested before on nets and have been found to be tolerant to human health. However, sneezing, runny nose, headache, numbness, itching, discharge from eyes, nausea, and unpleasant smell have been recorded in some people when new nets freshly taken from their package are used for the first time or few days of use. We will ask you for these symptoms, as well as any other adverse effects of using them. Although, most of these adverse effects will stop after a day or two. But, if symptoms persist for more than 48 hours, please consult a doctor at the local health facility or report to our staff immediately at the contact details given below and we will provide you with all the necessary medical care.

**Benefits**

ITENs with ITWS will protect you and everyone in your household from indoor mosquito bites and malaria. The information you will provide about the tools while using it will help the Institute, stakeholders and government to know the best tool to control malaria and other vector-borne diseases in your community, Tanzania or globally.

**Voluntary participation: right to refuse or withdraw consent**

Your participation in the study and interviews is entirely voluntary. You are not under any obligation to participate. If at any time during the study or interviews, you decide not to participate further, you are free to withdraw immediately, with no further discussion. This will have no adverse consequences on you. The study ITENs with ITWS that have been installed in your house belong to you and are yours to keep.

**Confidentiality**

All information related to your participation will be kept confidential and will not be revealed to anyone, except if required by law, such as in a legal request for the list of beneficiaries. Your identity will not be revealed in any reports or publications resulting from the study. The results of the interview will be put into a computer with the code numbers of the household, but without the names of the people interviewed. The data, both in hard copy and digital format, collected will be analysed to prepare a report for the London School of Hygiene and Tropical Medicine (LSHTM) and will be archived at IHI for scientific reference.

**Sharing of results**

The main outcomes of the study will be communicated to all villagers in a community meeting upon completion of the study. Any important new information concerning the results of our study will be made known to you.

**Who to contact?**

If you have any questions, you may ask them now or later. If you wish to ask questions later, you may contact any of the following, **they will provide answers to your questions**:

Zawadi Mageni. Mobile no.: +255 757177155

**Ms Rose Phillipo Mobile no.: +255 714583404.**

Should you wish to contact any of the above-named officials on phone, you need not spend your money but approach our village-level health worker who will facilitate the phone call on our behalf.

This proposal has been reviewed and approved by Ifakara Health Institute (IHI) Institutional Review Board and the National Institute of Medical Research (NIMR) Institutional Review Board, which are committees whose task are to make sure that research participants are protected from harm.

However, if you are not satisfied with responses given by the study team, feel free to contact the representative of IHI institutional review board **Dr. Mwifadhi Mrisho**, (+255 0788766676), or NatHREC Secretariat (0222121400) National Institute of Medical Research.

We are leaving with you a copy of this informed consent form for your information and future reference.

**PART 2. CERTIFICATE OF CONSENT**

*(This is an integral part of the information sheet and not a stand-alone document)*

*I have read the foregoing information, or it has been read to me in kiswahili. I have had the opportunity to ask questions about it and any questions that I have asked have been answered to my satisfaction. I consent voluntarily to participate as a householder in this study and understand that I have the right to withdraw from the study at any time without in any way affecting my medical care.* *I also understand that the Principal Investigator of the study can exclude my household from the study without my consent. I have been provided with a copy of this consent form.*

Participant Name : _____________________________________________

Participant Signature : ______________________Date ________________DD/MM/YY

***If illiterate***

*I have witnessed the accurate reading of the consent form to the potential participant, and the individual had the opportunity to ask questions. I confirm that the individual has given consent freely.*

Name of Independent Literate Witness: _____________________________________

Signature of Witness: ______________________ Date ________________DD/MM/YY

(If possible, this person should be selected by the participant and should have no connection to the research team)

**Statement by the researcher/person taking consent**

*I have accurately read or witnessed the accurate reading of the consent form to the potential participant, and the individual has had the opportunity to ask questions. I confirm that the individual has given consent freely.*

Name of Researcher: _____________________________________________

Signature of Researcher: ____________________ Date ________________DD/MM/YY

**INFORMED CONSENT FORM FOR HOUSEHOLDERS FOR FOLLOW-UP ITENs SURVEY**

**Name of Principle Investigator:** Dr. Zawadi Mageni Mboma

**Name of Organization:** Ifakara Health Institute, Bagamoyo, Tanzania

**Name of Sponsor:** London School of Hygiene and Tropical Medicine (LSHTM)

Household ID No. |__|__|__|__|

**Part 1: Information sheet for households**

**Feasibility and community acceptability of Insecticide Treated Eave Nets and Insecticide Treated Window screens in Tanzania.**

**Introduction**

My name is …………………...<name of ICF administrator>. I am working for the Ifakara Health Institute, Tanzania. We are here to investigate the attrition, fabric integrity and community acceptability of the Insecticide Treated Eave Nets (ITENs) with Insecticide Treated Window screens (ITWS) installed in your house. This study is meant to assess if ITENs and ITWS are still present and intact in your house.

**If you agree to participate in the study, you shall provide information about your household and we shall request for permission to come in your house to assess the condition of the ITENs and ITWS and ask questions about the condition.**

**If you agree to participate, below are the research procedures:**

1. Head or any adult member of your household will be interviewed and questions about your household’s demographic and socioeconomic characteristics, house structures, sleeping arrangement, pattern of use of nets and perception on ITENs with ITWS will be asked.
2. The team shall interview you and your household members on experiences with the presence of ITENs and ITWS in your house since installation.
3. The team shall check for the presence and condition of the ITENs with ITWS and you shall also be interviewed on acceptance of the tool, handling, use and perception of the tool.
4. The interview may last about 30 minutes.
5. In addition, you may also be selected to participate in a Focus Group Discussion about your experience on the use of ITENs and ITWS.
6. We may also cut a piece of net (25cm by 25 cm) from your windows and eaves facing the east and west to check if the nets are still killing mosquitoes in the laboratory, this will be repaired with new net.

**Benefits**

The information you will provide about the ITENs and ITWS while using it will help the study investigators, stakeholders and government to know the best tool to control malaria and other vector-borne diseases in your community, Tanzania and other malaria endemic countries.

**Voluntary participation: right to refuse or withdraw consent**

Your participation in the study and interviews is entirely voluntary. You are not under any obligation to participate. If at any time during the study or interviews, you decide not to participate further, you are free to withdraw immediately, with no further discussion. This will have no adverse consequences on you. The study ITENs and ITWS that have been installed in your house belong to you and are yours to keep.

**Confidentiality**

All information related to your participation will be kept confidential and will not be revealed to anyone, except if required by law, such as in a legal request for the list of beneficiaries. Your identity will not be revealed in any reports or publications resulting from the study. The results of the interview will be put into a computer with the code numbers of the household, but without the names of the people interviewed. The data, both in hard copy and digital format, collected will be analysed to prepare a report for the London School of Hygiene and Tropical Medicine (LSHTM) and will be archived at IHI for scientific reference.

**Sharing of results**

The main outcomes of the study will be communicated to all villagers in a community meeting upon completion of the study. Any important new information concerning the results of our study will be made known to you.

**Who to contact?**

If you have any questions or queries you may ask them now or later. If you wish to ask questions later, you may contact any of the following,

**Zawadi Mageni Mboma**: Mobile no.: +**255 787428218**

**Ms Rose Phillipo Mobile no.: +255 714583404.**

Should you wish to contact any of the above-named officials on phone, you need not spend your money but approach our village-level head who will facilitate the phone call on our behalf.

This proposal has been reviewed and approved by Ifakara Health Institute (IHI) Institutional Review Board and the National Institute of Medical Research (NIMR) Institutional Review Board, which are committees whose task are to make sure that research participants are protected.

However, if you are not satisfied with responses given by the study team, feel free to contact the representative of IHI institutional review board **Dr. Mwifadhi Mrisho**, (+255 0788766676), or NatHREC Secretariat (0222121400) National Institute of Medical Research.

A copy of this informed consent form will be left for your information and future reference.

**PART 2. CERTIFICATE OF CONSENT**

*(This is an integral part of the information sheet and not a stand-alone document)*

*I have read the foregoing information, or it has been read to me in kiswahili. I have had the opportunity to ask questions about it and any questions that I have asked have been answered to my satisfaction. I consent voluntarily to participate as a householder in this study and understand that I have the right to withdraw from the study at any time without in any way affecting my medical care.* *I also understand that the Principal Investigator of the study can exclude my household from the study without my consent. I have been provided with a copy of this consent form.*

Participant Name : _____________________________________________

Participant Signature : ______________________Date ________________DD/MM/YY

***If illiterate***

*I have witnessed the accurate reading of the consent form to the potential participant, and the individual had the opportunity to ask questions. I confirm that the individual has given consent freely.*

Name of Independent Literate Witness: _____________________________________

Signature of Witness: ______________________ Date ________________DD/MM/YY

(This person should be selected by the participant and should have no connection to the research team)

**Statement by the researcher/person taking consent**

*I have accurately read or witnessed the accurate reading of the consent form to the potential participant, and the individual has had the opportunity to ask questions. I confirm that the individual has given consent freely.*

Name of Researcher: _____________________________________________

Signature of Researcher: ____________________ Date ________________DD/MM/YY

**INFORMED CONSENT FORM FOR AN ADULT FOR MALARIA TESTING**

**Name of Principle Investigator:** Dr. Zawadi Mageni Mboma

**Name of Organization:** Ifakara Health Institute, Bagamoyo, Tanzania

**Name of Sponsor:** London School of Hygiene and Tropical Medicine (LSHTM)

Household ID No. |__|__|__|__|

**Part 1: Information sheet for households**

**Feasibility and community acceptability of Insecticide Treated Eave Nets and Insecticide Treated Window screens in Tanzania.**

**Introduction**

My name is …………………...<name of ICF administrator>. I am working for the Ifakara Health Institute, Tanzania. We are here to investigate the effectiveness of Insecticide Treated Eave Nets (ITENs) with Insecticide Treated Window screens (ITWS) on malaria. This study is to check if the nets protect from malaria.

If you agree to participate in the study, we shall ask for your demographic information and the children in your household. We shall conduct malaria test and fever checks on you. We shall also request for your consent to conduct malaria tests and fever checking on the children in your household. Two tests will be done, that include, malaria Rapid Diagnostic Test (mRDT) and dry blood spots. All these tests will involve pricking of a finger. The pricking for all these tests will only be done once. The blood will be stored for 10 years at IHI, Bagamoyo for malaria testing only, and will not be used for another test.

**If you agree to participate, below are the research procedures:**

1. You shall provide your demographic information and that of your children.
2. We shall conduct malaria test on you by pricking your finger to take blood for mRDT and storage to check for malaria in the laboratory located in IHI, Bagamoyo.
3. We shall check you for fever using body thermometer.
4. We shall also conduct malaria and fever check on your children above 6 months old.
5. The procedure may last about 20 minutes.

**Risks**

There is no risk in pricking your finger because the kits are new and withdrawal of no more than 500µl of blood will be done. The only risk is a slight physical pain from pricking the finger when performing the screening test.

**Benefits**

If you or your children test positive for malaria, treatment will be offered.

**Voluntary participation: right to refuse or withdraw consent**

Your participation in the study and interviews is entirely voluntary. You are not under any obligation to participate. If at any time during the study or interviews, you decide not to participate further, you are free to withdraw immediately, with no further discussion. This will have no adverse consequences on you. The study ITENs with ITWS that have been installed in your house belong to you and are yours to keep.

**Confidentiality**

All information related to your participation will be kept confidential and will not be revealed to anyone, except if required by law, such as in a legal request for the list of beneficiaries. Your identity will not be revealed in any reports or publications resulting from the study. The results of the interview will be put into a computer with the code numbers of the household, but without the names of the people interviewed. The data, both in hard copy and digital format, collected will be analysed to prepare a report for the London School of Hygiene and Tropical Medicine (LSHTM) and will be archived at IHI for scientific reference.

**Sharing of results**

The main outcomes of the study will be communicated to all villagers in a community meeting upon completion of the study. Any important new information concerning the results of our study will be made known to you.

**Who to contact?**

If you have any questions or queries you may ask them now or later. If you wish to ask questions later, you may contact any of the following,

**Zawadi Mageni Mboma**: Mobile no.: +**255 787428218**

**Ms Rose Phillipo Mobile no.: +255 714583404.**

Should you wish to contact any of the above-named officials on phone, you need not spend your money but approach our village-level health worker who will facilitate the phone call on our behalf.

This proposal has been reviewed and approved by Ifakara Health Institute (IHI) Institutional Review Board and the National Institute of Medical Research (NIMR) Institutional Review Board, which are committees whose task are to make sure that research participants are protected from harm.

However, if you are not satisfied with responses given by the study team, feel free to contact the representative of IHI institutional review board **Dr. Mwifadhi Mrisho**, (+255 0788766676), or NatHREC Secretariat (0222121400) National Institute of Medical Research.

A copy of this informed consent form will be left for your information and future reference.

**PART 2. CERTIFICATE OF CONSENT**

*(This is an integral part of the information sheet and not a stand-alone document)*

*I have read the foregoing information, or it has been read to me in kiswahili. I have had the opportunity to ask questions about it and any questions that I have asked have been answered to my satisfaction. I consent voluntarily to participate as a householder in this study and understand that I have the right to withdraw from the study at any time without in any way affecting my medical care.* *I also understand that the Principal Investigator of the study can exclude my household from the study without my consent. I have been provided with a copy of this consent form.*

Participant Name : _____________________________________________

Participant Signature : ______________________Date ________________DD/MM/YY

***If illiterate***

*I have witnessed the accurate reading of the consent form to the potential participant, and the individual had the opportunity to ask questions. I confirm that the individual has given consent freely.*

Name of Independent Literate Witness: _____________________________________

Signature of Witness: ______________________ Date ________________DD/MM/YY

(If possible, this person should be selected by the participant and should have no connection to the research team)

**Statement by the researcher/person taking consent**

*I have accurately read or witnessed the accurate reading of the consent form to the potential participant, and the individual has had the opportunity to ask questions. I confirm that the individual has given consent freely.*

Name of Researcher: _____________________________________________

Signature of Researcher: ____________________ Date ________________DD/MM/YY

**ADOLESCENT (Ages 13-17) ASSENT TO PARTICIPATE IN MALARIA TESTING**

**Name of Principle Investigator:** Dr. Zawadi Mageni Mboma

**Name of Organization:** Ifakara Health Institute, Bagamoyo, Tanzania

**Name of Sponsor:** London School of Hygiene and Tropical Medicine (LSHTM)

Household ID No. |__|__|__|__|

**Part 1: Information sheet for households**

**Feasibility and community acceptability of Insecticide Treated Eave Nets and Insecticide Treated Window screens in Tanzania.**

**Introduction**

My name is …………………...<name of ICF administrator>. I am working for the Ifakara Health Institute, Tanzania. We are here to investigate the effect of Insecticide Treated Eave Nets (ITENs) with Insecticide Treated Window screens (ITWS) on malaria. These nets were made basically not to need retreatment throughout their functional life, the insecticide in them kills mosquitoes and the nets block all mosquito from entry. Therefore, we want to check if it can prevent people from malaria.

You have been selected to participate in this study. Please talk this over with your parents before you decide whether or not to participate. We will also ask your parents to give their permission for you to take part in this study. But even if your parents say “yes” you can still decide not to do this.

If you agree to be in this study, we shall conduct malaria tests on you and we will also check if you have fever using thermometer. Two tests will be done, that include, RDT and dry blood spots. All these tests will involve pricking of a finger. The pricking for all these tests will only be done once.

There are NO health risks associated with this particular study. The only risk is a slight physical pain from pricking the finger when performing the screening test. If you are found to be malaria positive, we will provide malaria medication for you.

**Voluntary participation: right to refuse or withdraw consent**

Your participation in the study and interviews is entirely voluntary. You are not under any obligation to participate. If at any time during the study, you decide not to participate further, you are free to withdraw immediately, with no further discussion. This will have no adverse consequences on you.

**Confidentiality**

All information related to your participation will be kept confidential and will not be revealed to anyone, except if required by law, such as in a legal request for the list of beneficiaries. Your identity will not be revealed in any reports or publications resulting from the study. The results of the interview will be put into a computer with the code numbers of the household, but without the names of the people interviewed. The data, both in hard copy and digital format, collected will be analysed to prepare a report for the London School of Hygiene and Tropical Medicine (LSHTM) and will be archived at IHI for scientific reference.

**Sharing of results**

The main outcomes of the study will be communicated to all villagers in a community meeting upon completion of the study. Any important new information concerning the results of our study will be made known to you.

**Who to contact?**

If you have any questions or queries you may ask them now or later. If you wish to ask questions later, you may contact any of the following,

**Zawadi Mageni Mboma**: Mobile no.: +**255 787428218**

**Ms Rose Phillipo Mobile no.: +255 714583404.**

Should you wish to contact any of the above-named officials on phone, you need not spend your money but approach our village-level health worker who will facilitate the phone call on our behalf.

This proposal has been reviewed and approved by Ifakara Health Institute (IHI) Institutional Review Board and the National Institute of Medical Research (NIMR) Institutional Review Board, which are committees whose task are to make sure that research participants are protected from harm.

However, if you are not satisfied with responses given by the study team, feel free to contact the representative of IHI institutional review board **Dr. Mwifadhi Mrisho**, (+255 0788766676), or NatHREC Secretariat (0222121400) National Institute of Medical Research.

A copy of this informed consent form will be left for your information and future reference.

**SIGNATURE OF STUDY PARTICIPANT**

I understand the procedures described above. My questions have been answered to my satisfaction, and I agree to participate in this study. I have been given a copy of this form.

|  |  |  |
| --- | --- | --- |
| Name of Participant |  |  |

|  |  |  |
| --- | --- | --- |
| Signature of Participant |  | Date |

**SIGNATURE OF PERSON OBTAINING ASSENT**

In my judgment the participant is voluntarily and knowingly agreeing to participate in this research study.

|  |  |  |
| --- | --- | --- |
| Name of Person Obtaining Assent |  | Contact Number |

|  |  |  |
| --- | --- | --- |
| Signature of Person Obtaining Assent |  | Date |

**ASSENT FOR CHILDREN 7-12 YEARS OLD TO PARTICIPATE IN MALARIA TESTING**

**Name of Principle Investigator:** Dr. Zawadi Mageni Mboma

**Name of Organization:** Ifakara Health Institute, Bagamoyo, Tanzania

**Name of Sponsor:** London School of Hygiene and Tropical Medicine (LSHTM)

Household ID No. |__|__|__|__|

**Feasibility and community acceptability of Insecticide Treated Eave Nets and Insecticide Treated Window screens in Tanzania.**

1. My name is […………………………………], I am working for the Ifakara Health Institute, Tanzania.
2. We are here to investigate the effectiveness of Insecticide Treated Eave Nets (ITENs) with Insecticide Treated Window screens (ITWS) installed in your house. These nets were made basically not to need retreatment throughout their functional life, the insecticide in them kills mosquitoes and the nets block all mosquito from entry. Therefore, we want to check if it can prevent people from malaria.
3. If you agree to be in this study, you will be requested to do malaria tests and test if you have fever. Two tests will be done, that include, RDT and dry blood spots. All these tests will involve pricking of a finger. The pricking for all these tests will only be done once.
4. There are **NO** health risks associated with this particular study. The only risk is a slight physical pain from pricking the finger when performing the screening test.
5. There will be no direct benefits towards you but your participation will help us determine exact incidence of malaria in your community. Through this information, we will be able to know if ITENs control malaria.
6. Please talk this over with your parents before you decide whether or not to participate. We will also ask your parents to give their permission for you to take part in this study. But even if your parents say “yes” you can still decide not to do this.
7. If you don’t want to be in this study, you don’t have to participate. Remember, being in this study is up to you and no one will be upset if you don’t want to participate or even if you change your mind later and want to stop.
8. You can ask any questions that you have about the study. If you have a question later that you didn’t think of now, you can call either of the two numbers below or ask me next time:

**Dr Zawadi Mageni Mboma: Phone number: +255 0787428218**

**Ms Rose Phillipo – Phone number: +255 714583404**

1. Signing your name at the bottom means that you agree to be in this study. In case you are found with malaria, you will be provided with ALU free of charge. You and your parents will be given a copy of this form after you have signed it.

________________________________________ ____________________

Name of participant Date

**INFORMED CONSENT FORM FOR MOSQUITO DENSITY SURVEY**

**Name of Principle Investigator:** Dr. Zawadi Mageni Mboma

**Name of Organization:** Ifakara Health Institute, Bagamoyo, Tanzania

**Name of Sponsor:** London School of Hygiene and Tropical Medicine (LSHTM)

Household ID No. |__|__|__|__|

**Part 1: Information sheet for households**

**Feasibility and community acceptability of Insecticide Treated Eave Nets and Insecticide Treated Window screens in Tanzania.**

**Introduction**

My name is …………………...<name of ICF administrator>. I am working for the Ifakara Health Institute, Tanzania. We are here to investigate the effect of Insecticide Treated Eave Nets (ITENs) with Insecticide Treated Window screens (ITWS) on mosquito density inside the house. This study is meant to know if the nets are truly preventing the mosquitoes from indoor entry.

**If you agree to participate in the study, we shall install CDC light traps in your house from 18:00 to 6:00.**

**If you agree to participate, below are the research procedures:**

1. Field team will seek for your permission to install Center for Disease Control (CDC) light traps in your house.
2. One CDC light trap will be placed at one of the sleeping spaces used in the household at the foot end of the bed, with the light source positioned at approximately 0.7m from the ground.
3. Field team will install the trap at 18:00 and collect the trap in the morning at 6:00.
4. This will only be done in your house once.

**Risks and adverse effects**

Installation of CDC light trap in your house do not have any risk except from the inconvenience of coming to your house in the evening and morning when you may be sleeping.

**Benefits**

CDC light traps will catch some of the mosquitoes that are supposed to bite you, thereby giving you and your family overnight protection from mosquitoes.

**Voluntary participation: right to refuse or withdraw consent**

Your participation in the study and interviews is entirely voluntary. You are not under any obligation to participate. If at any time during the study or interviews, you decide not to participate further, you are free to withdraw immediately, with no further discussion. This will have no adverse consequences on you. The study ITENs with ITWS that have been installed in your house belong to you and are yours to keep.

**Confidentiality**

All information related to your participation will be kept confidential and will not be revealed to anyone, except if required by law, such as in a legal request for the list of beneficiaries. Your identity will not be revealed in any reports or publications resulting from the study. The results of the interview will be put into a computer with the code numbers of the household, but without the names of the people interviewed. The data, both in hard copy and digital format, collected will be analysed to prepare a report for the London School of Hygiene and Tropical Medicine (LSHTM) and will be archived at IHI for scientific reference.

**Sharing of results**

The main outcomes of the study will be communicated to all villagers in a community meeting upon completion of the study. Any important new information concerning the results of our study will be made known to you.

**Who to contact?**

If you have any questions or queries you may ask them now or later. If you wish to ask questions later, you may contact any of the following,

**Zawadi Mageni Mboma**: Mobile no.: +**255 787428218**

**Ms Rose Phillipo Mobile no.: +255 714583404.**

Should you wish to contact any of the above-named officials on phone, you need not spend your money but approach our village-level health worker who will facilitate the phone call on our behalf.

This proposal has been reviewed and approved by Ifakara Health Institute (IHI) Institutional Review Board and the National Institute of Medical Research (NIMR) Institutional Review Board, which are committees whose task are to make sure that research participants are protected from harm.

However, if you are not satisfied with responses given by the study team, feel free to contact the representative of IHI institutional review board **Dr. Mwifadhi Mrisho**, (+255 0788766676), or NatHREC Secretariat (0222121400) National Institute of Medical Research.

A copy of this informed consent form will be left for your information and future reference.

**PART 2. CERTIFICATE OF CONSENT**

*(This is an integral part of the information sheet and not a stand-alone document)*

*I have read the foregoing information, or it has been read to me in kiswahili. I have had the opportunity to ask questions about it and any questions that I have asked have been answered to my satisfaction. I consent voluntarily to participate as a householder in this study and understand that I have the right to withdraw from the study at any time without in any way affecting my medical care.* *I also understand that the Principal Investigator of the study can exclude my household from the study without my consent. I have been provided with a copy of this consent form.*

Participant Name : _____________________________________________

Participant Signature : ______________________Date ________________DD/MM/YY

***If illiterate***

*I have witnessed the accurate reading of the consent form to the potential participant, and the individual had the opportunity to ask questions. I confirm that the individual has given consent freely.*

Name of Independent Literate Witness: _____________________________________

Signature of Witness: ______________________ Date ________________DD/MM/YY

(if possible, this person should be selected by the participant and should have no connection to the research team)

**Statement by the researcher/person taking consent**

*I have accurately read or witnessed the accurate reading of the consent form to the potential participant, and the individual has had the opportunity to ask questions. I confirm that the individual has given consent freely.*

Name of Researcher: _____________________________________________

Signature of Researcher: ____________________ Date ________________DD/MM/YY
